# Reliability modelling and analysis of a multi-state element based on a dynamic Bayesian network

**DOI:** 10.1098/rsos.171438

**Published:** 2018-04-11

**Authors:** Zhiqiang Li, Tingxue Xu, Junyuan Gu, Qi Dong, Linyu Fu

**Affiliations:** Naval Aeronautical University, Shandong 264001, People's Republic of China

**Keywords:** multi-state element, Markov process, dynamic Bayesian network, condition-based maintenance, conditional probability table, dynamic fault tree

## Abstract

This paper presents a quantitative reliability modelling and analysis method for multi-state elements based on a combination of the Markov process and a dynamic Bayesian network (DBN), taking perfect repair, imperfect repair and condition-based maintenance (CBM) into consideration. The Markov models of elements without repair and under CBM are established, and an absorbing set is introduced to determine the reliability of the repairable element. According to the state-transition relations between the states determined by the Markov process, a DBN model is built. In addition, its parameters for series and parallel systems, namely, conditional probability tables, can be calculated by referring to the conditional degradation probabilities. Finally, the power of a control unit in a failure model is used as an example. A dynamic fault tree (DFT) is translated into a Bayesian network model, and subsequently extended to a DBN. The results show the state probabilities of an element and the system without repair, with perfect and imperfect repair, and under CBM, with an absorbing set plotted by differential equations and verified. Through referring forward, the reliability value of the control unit is determined in different kinds of modes. Finally, weak nodes are noted in the control unit.

## 1. Introduction

The reliability of a system or an element is defined as: the ability to perform its required functions under specific operating conditions for a specified period of time [1]. Traditional analysis methods, such as a fault tree analysis (FTA), a binary decision diagram (BDD) and a failure modes and effects analysis (FMEA), are suggested for the purpose of the reliability evaluation. When applying the FTA, BDD or FMEA, assumptions are made that there are only two states in the system, normal and failure, and the events in the system are independent of each other. However, in real-world systems, in addition to perfect functionality and complete failure, an element may have several intermediate states; therefore, it is considered a multi-state element (MSE). A system consisting of MSEs is called a multi-state system (MSS). In addition, as redundant design and dynamic logic gates are introduced, systems become more complex and sophisticated, and traditional analysis methods no longer apply. Thus, new methods are required to assess the reliability parameters from the perspective of multi-states or multi-stages to decrease the downtime probability and degradation of complex systems [2,3].

To determine the dynamic characteristic parameters of MSEs or single MSE systems, many multi-state models have been established based on Markov processes in the domains of engineering, medicine and economics [4–6]. The Markov processes are widely used because the number of failures in arbitrary time intervals can be described as a Poisson process, and the corresponding time to failure and repair are assumed to obey an exponential distribution. Anatoly *et al*. [4] built a multi-state Markov model to predict the reliability of a coal power generating unit for a short-term range. Viewing the disease process as a multi-state progression, Malcolm *et al*. [5] performed a meta-analysis to determine the parameters of the treatment effects in multi-state Markov models. Similarly, Azza & Adel [6] extended the Markov-switching model to build a four-state indicator to detect inflexions and deterioration. When transition densities of MSEs between states do not obey exponential distributions, modified Markov models are applied to describe the degradation and maintenance process of MSEs including perfect repair, minimal repair and imperfect repair [7–9].

To obtain the reliability parameters of an MSS, Helge & Luigi [10] applied a Bayesian network (BN) in the reliability analysis community, and discussed its relevant ongoing research for practitioners. The BN was developed on the basis of probability and graph theory, and it is advantageous for performing a forward or predictive analysis and backward or diagnostic analysis, and for expressing uncertain causal relations [11,12]; the BN is widely used in system reliability assessment [13–15], human reliability analysis [16,17], fusing uncertain information [18,19] and operational risk assessment [20,21]. The BN can describe any MSE or MSS with a single node, which simplifies the state-transition in the stochastic process. In addition, all causal relationships can be denoted by conditional probability distributions. For deterministic logic relations, the conditional probability tables (CPTs) can be obtained through static or dynamic logic gates. In other cases, the CPTs can be obtained by consulting experts or referring to recorded failure data.

Methods such as FTA, BDD and FMEA are static tools used to direct the reliability improvement of the system or its elements at the beginning or at a specific time. A dynamic fault tree (DFT) is developed on the basis of a Markov process and is a useful tool to expand and upgrade the existing models to further improve the reliability and reduce system unavailability [22–24]. Because of the state explosion problem in Markov processes and the difficulty in obtaining a minimal cut sequence set, the DFT application is limited in complex systems with many dynamic logic gates. By introducing relevant temporal dependencies between representations, a BN is expanded into a dynamic Bayesian network (DBN), which overcomes the shortcomings of a DFT [21,25]. Compared with a DFT, a DBN is more suitable for monitoring and predicting the change of random variables and representing states of the system or its elements at any time. Daniele *et al*. [26] reported a DBN framework inside a system or among systems to evaluate cascading effects in a power grid. Shubharthi *et al*. [20] mapped a DFT into a DBN to perform a dynamic operational risk assessment and illustrated the methodological capability. Esmaeil *et al*. [27] developed a DBN model for an accident scenario and the risk associated with natural gas stations and indicated the failure of a regulator system.

In a reliability analysis, repair is a non-negligible factor. Fan *et al*. [28] introduced an algorithm based on a DBN for a repairable model to evaluate the reliability and security of complex systems. Cai & Liu [29,30] developed a reliability model of subsea blowout preventers to perform a common cause failure analysis based on a DBN. To improve the benefit of combined maintenance, Wang *et al*. [31] established a stochastic deterioration model for multi-element systems under condition-based maintenance (CBM). For equipment inaccessible to humans, repairs, including perfect repairs and imperfect repairs after a failure, are adopted. For equipment under monitoring, the CBM is better. When degradation or a failure occurs, maintenance measures can be adopted immediately. This paper is structured as follows: §2 presents the reliability model of an MSE based on Markov processes; §3 illustrates the method to develop a DBN of MSEs; §4 illustrates a control unit as an example; the results and discussion are considered in §5; and §6 summarizes this paper (table 1).

**Table 1.** Nomenclature.

*k*	number of performance states
*g_i_*	performance rate of an element in state *i*
*G*(*t*)	performance rate of an element at time *t*
*p_i_*(*t*)	probability of an element in state *i* at time *t*
*w_i_*	desired performance at level *i*
*W*(*t*)	desired level of performance at time *t*
*F*(*G*(*t*), *W*(*t*))	acceptability function between the performance and demand
*λ_e_*_,*i*_	degradation intensity from state *e* to state *i*
*R_i_*(*t*)	reliability function of an element with performance rate higher than *g_i_*
*μ_i_*_,*e*_	repair intensity from state *i* to state *e*
*B*_1_	initial Bayesian network
$B_{\to }$	Bayesian networks including multiple copies of time slices
*P*(*X_t_*|*X_t_*_−1_)	transition probability between two adjacent time slices
$X_{t}^{i}$	the *i*th node at time slice *t*
$pa(X_{t}^{i})$	the parent nodes of the *i*th node at time slice *t*
Δ*t*	time interval between two consecutive time slices at any time *t*
*f_j_*	degradation probability of node *j*
*P*(*Y*|*X*_1_, *X*_2_, … , *X_n_*)	unreliability function for logic gates with conditional degradation probabilities

## 2. Reliability modelling of a multi-state element

An element has *k* different states corresponding to its performance rates, denoted by the set *g*  =  {*g*_1_, *g*_2_, … , *g_k_*}, *g_i_*_+1_ > *g_i_* for any *i*. Herein, *g_k_* represents the perfect functionality state of the element, and *g*_1_ represents the complete failure state. The intermediate value *g_i_*(1 < *i* < *k*) denotes a state of degradation. At any time, the performance rate *G*(*t*) of an element is a random variable taking a value from *g*, resulting in *G*(*t*) ∈ *g*. Assume that *p*(*t*)  =  {*p*_1_(*t*), *p*_2_(*t*), … , *p_k_*(*t*)} is the probability set associated with different states of the element at any time *t*. Now that *g* concludes the complete group of exclusive events, then $\sum_{i=1}^{k}p_{i}(t)=1$ for any *t*:0  ≤  *t*  ≤  *T*. Assume that the desired level of performance *W*(*t*) takes discrete values from a set *w*  =  {*w*_1_, *w*_2_, … , *w_m_*}. The acceptability function *F*(*G*(*t*), *W*(*t*)) expresses the desired relationship between the performance and demand. If *F*(*G*(*t*), *W*(*t*))  ≥  0, it refers to the acceptable states, and if *F*(*G*(*t*), *W*(*t*)) < 0, it refers to the unacceptable states defined as failures. The MSEs are divided into two groups, non-repairable elements and repairable elements.

### 2.1. Modelling of non-repairable elements

**Figure 1.**
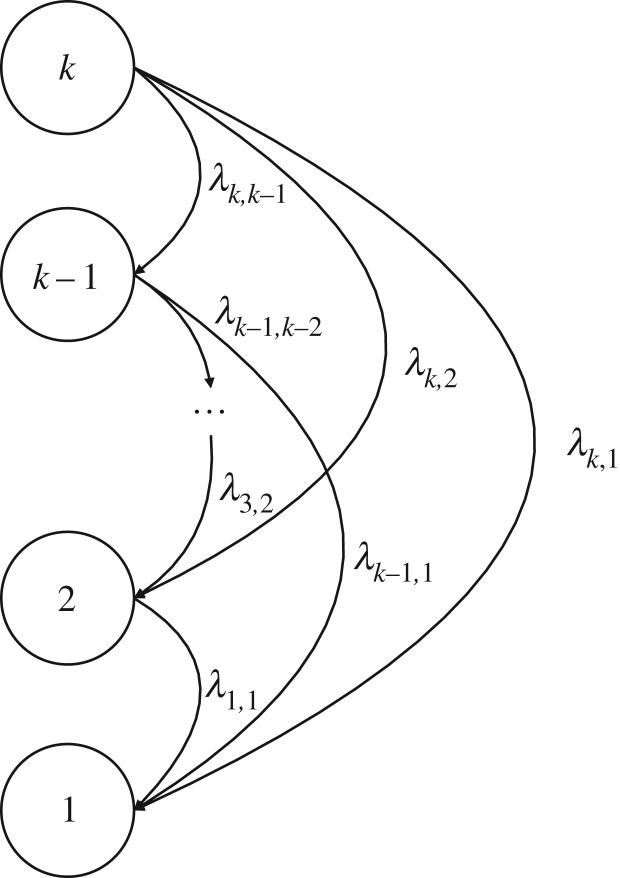
State-transition diagram for a non-repairable element.

The case where an MSE can enter the subset only once usually refers to a non-repairable deteriorating element. The element acceptability depends on the relation between the element performance and the desired demand. An MSE has two kinds of failures: minor failures and major failures, which can occur at any time. Minor failures cause an element transition from state *i* to the adjacent state *i* − 1, while major failures cause an element transition from state *i* to state *j*:*j* < *i* − 1. Assume that the sojourn time in any state is exponentially distributed. The state transition diagram is presented in figure 1. In addition, the corresponding differential equations are written as follows to find the state probabilities for the Markov process.

2.1 $$\begin{array}{rl} & \frac{\text{d}p_{k}(t)}{\text{d}t}=-p_{k}(t)\sum_{e=1}^{k-1}\lambda_{k,e,}\\ & \frac{\text{d}p_{i}(t)}{\text{d}t}=\sum_{e=i+1}^{k}\lambda_{e,i}p_{e}(t)-p_{i}(t)\sum_{e=1}^{i-1}\lambda_{i,e},i=2,3,\cdots ,k-1,\\ & \frac{\text{d}p_{1}(t)}{\text{d}t}=\sum_{e=2}^{k}\lambda_{e,1}p_{e}(t), \end{array}\}$$

where *λ_e_*_,*i*_ represents the degradation intensity from state *e* to state *i*.

It is obvious that in state *k* there are *k* − 1 transitions from this state to state *e*:1  ≤  *e*  ≤  *k* − 1 with the intensity *λ_k_*_,*e*_, and there are no transitions back to state *k*. In each state *i*:2  ≤  *i*  ≤  *k* − 1, there are transitions to this state from upper states and transitions from this state to lower states. There are no transitions from state 1, which means it is an absorbing state for non-repairable MSEs.

At the very beginning, an element is in the best state *k* with a maximal performance rate of *g_k_*. Therefore, the initial conditions are

2.2 $$p_{k}(0)=1,p_{k-1}(0)=p_{k-2}(0)=\cdots =p_{1}(0)=0.$$

If the demand is *g_i_* < *w*  ≤  *g_i_*_+1_, *i*  =  1, 2, … , *k* − 1, the reliability function is denoted as

2.3 $$R_{i}(t)=1-\sum_{j=1}^{i}p_{j}(t)=\sum_{j=i+1}^{k}p_{j}(t).$$

### 2.2. Modelling of repairable elements

**Figure 2.**
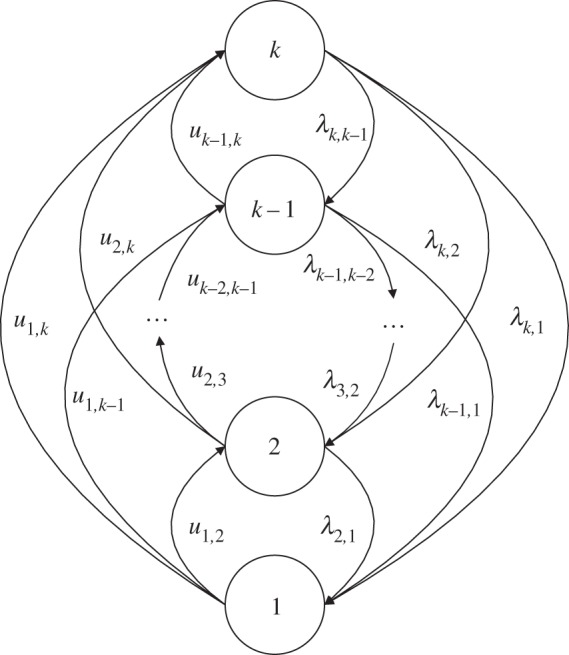
State-transition diagram for a repairable element.

For repairable elements, the transitions between subsets of acceptable states and unacceptable states can occur at any time. Similar to failures, repairs can be divided into two groups: minor repairs and major repairs. Minor repairs return an element from state *j* to the adjacent state *j* + 1 with the parameter *u_j_*_,*j*+1_, while major repairs return an element from state *j* to state *i*:*j* + 1 < *i* with the parameter *u_j_*_,*i*_. The differential equations are written as follows for the state probabilities for the repairable MSE with minor and major failures and repairs, as shown in figure 2.

2.4 $$\begin{array}{rl} & \frac{\text{d}p_{k}(t)}{\text{d}t}=\sum_{e=1}^{k-1}u_{e,k}p_{e}(t)-p_{k}(t)\sum_{e=1}^{k-1}\lambda_{k,e},\\ & \frac{\text{d}p_{i}(t)}{\text{d}t}=\sum_{e=i+1}^{k}\lambda_{e,i}p_{e}(t)+\sum_{e=1}^{i-1}u_{e,i}p_{e}(t)-p_{i}(t)\left(\sum_{e=1}^{i-1}\lambda_{i,e}+\sum_{e=i+1}^{k}u_{i,e}\right),i=2,3,\cdots ,k-1,\\ & \frac{\text{d}p_{1}(t)}{\text{d}t}=\sum_{e=2}^{k}\lambda_{e,1}p_{e}(t)-p_{1}(t)\sum_{e=2}^{k}u_{1,e}, \end{array}\}$$

where *μ_i_*_,*e*_ represents the repair intensity from state *i* to state *e*.

In addition, the initial conditions are the same as those for equation (2.2).

**Figure 3.**
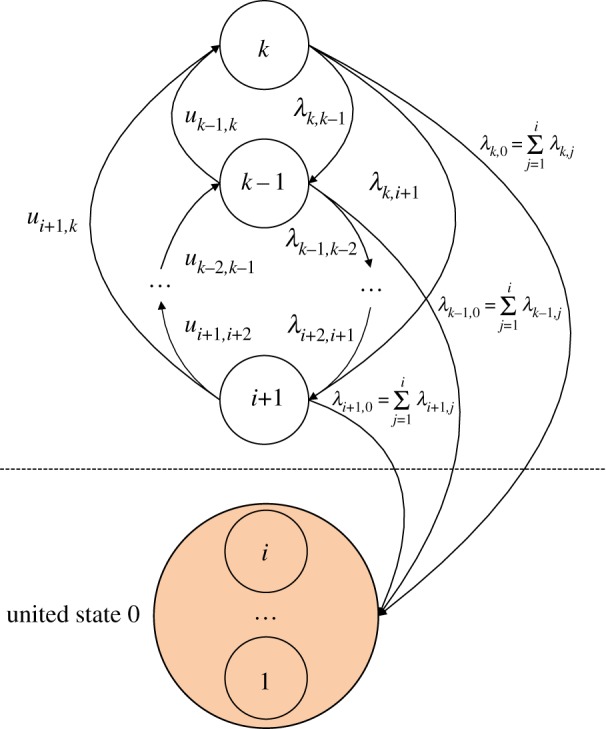
State-transition diagram for a repairable element under a constant demand.

To determine the reliability function for repairable MSEs, the probability of the element entering the set of unacceptable states for the first time must be obtained. To find the reliability function *R_i_*(*t*) for a constant demand *w*(*g_i_* < *w*  ≤  *g_i_*_+1_), another Markov model is established, as shown in figure 3. All states lower than the demand *w* are eliminated in an absorbing state, denoted as state 0. All repairs from this state back to acceptable states are forbidden, i.e. zeroing all the transition intensities *u*_0,*m*_ for *m*  =  *i*  +  1, · · · ,*k*. In addition, the transition intensity *λ_m_*_,0_ from any acceptable state *m* to state 0 is equal to that of the transitions to all the unacceptable states, denoted as

2.5 $$\lambda_{m,0}=\sum_{j=1}^{i}\lambda_{m,j},m=k,k-1,\cdots ,i+1.$$

The differential equations to determine the reliability of the repairable element are denoted as

2.6 $$\begin{array}{rl} & \frac{\text{d}p_{k}(t)}{\text{d}t}=\sum_{e=i+1}^{k-1}u_{e,k}p_{e}(t)-p_{k}(t)\left(\sum_{e=i+1}^{k-1}\lambda_{k,e}+\lambda_{k,0}\right),\\ & \frac{\text{d}p_{j}(t)}{\text{d}t}=\sum_{e=j+1}^{k}\lambda_{e,j}p_{e}(t)+\sum_{e=i+1}^{j-1}u_{e,j}p_{e}(t)-p_{j}(t)\left(\sum_{e=i+1}^{j-1}\lambda_{j,e}+\lambda_{j,0}+\sum_{e=j+1}^{k}u_{j,e}\right),i<j<k,\\ & \frac{\text{d}p_{0}(t)}{\text{d}t}=\sum_{e=i+1}^{k}\lambda_{e,0}p_{e}(t), \end{array}\}$$

with the initial conditions of equation (2.2), and the reliability function is obtained by equation (2.3). When *t* → ∞, the element enters state 0 with final state probabilities given by

2.7 $$p_{k}=p_{k-1}=\cdots =p_{i+1}=0,p_{0}=1.$$

## 3. Dynamic Bayesian network modelling for a multi-state element

### 3.1. Dynamic Bayesian network model

A DBN is an extension of the static BN by introducing the temporal evolution of variables. The DBN is represented as a pair (*B*_1_, *B*_→_), where *B*_1_ is the initial BN that defines the prior *P*(*X_t_*), and $B_{\to }$ are BNs that include multiple copies of time slices. The transition probability *P*(*X_t_*|*X_t_*_−1_) between two adjacent slices is

3.1 $$P(X_{t}|X_{t-1})=\prod_{i=1}^{N}P(X_{t}^{i}|pa(X_{t}^{i})).$$

Here, $X_{t}^{i}$ denotes the *i*th node at time slice *t*, and $pa(X_{t}^{i})$ denotes its parent nodes.

There are two assumptions in a DBN, i.e. the system is the first-order Markov and a time-homogeneous system. Therefore, the edges between the nodes in a DBN locate in the same slice or two adjacent slices. In addition, the parameters of the conditional probability distribution will not change as time progresses. By unrolling *T* time slices, the joint distribution probability is obtained by

3.2 $$P(X_{1:T})=\prod_{i=1}^{T}\prod_{i=1}^{N}P(X_{t}^{i}|pa(X_{t}^{i})).$$

**Figure 4.**
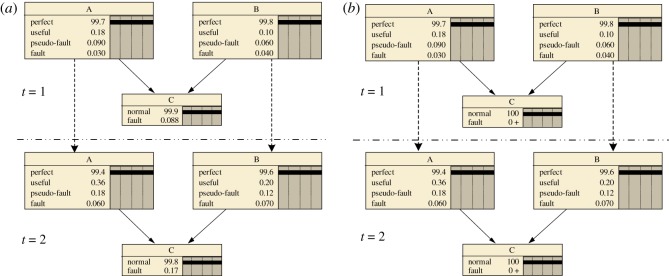
DBN model for (*a*) series and (*b*) parallel systems with two elements.

Shown in figure 4, the series and parallel systems are extended from time slice *t*  =  1 to *t*  =  2, respectively. In the series system shown in figure 4*a*, the nodes A and B at time *t*  =  1 are extended to time *t*  =  2 with an inter-slice arc, respectively. There is no intra-arc between nodes A and B, so they are independent of each other. The parent nodes A and B have four states, namely, the perfect, useful, pseudo-fault and fault states. The child node C has two states, namely, the normal and fault states. Having the same structure, except for different CPTs, the parallel system shown in figure 4*b* has a higher reliability value than the series system at time *t*  =  1 and *t*  =  2.

### 3.2. Dynamic Bayesian network modelling for a multi-state element

For a multi-state degraded element, four assumptions are described as follows:

(1) The element has many levels of degradation, taking a value from perfect functioning to a complete failure;
(2) The element may fail randomly at any time from operational states including minor failures and major failures;
(3) All state-transition rates are constant, obeying the exponential distribution;
(4) The current state of an element is observable through some testing parameters.

**Figure 5.**
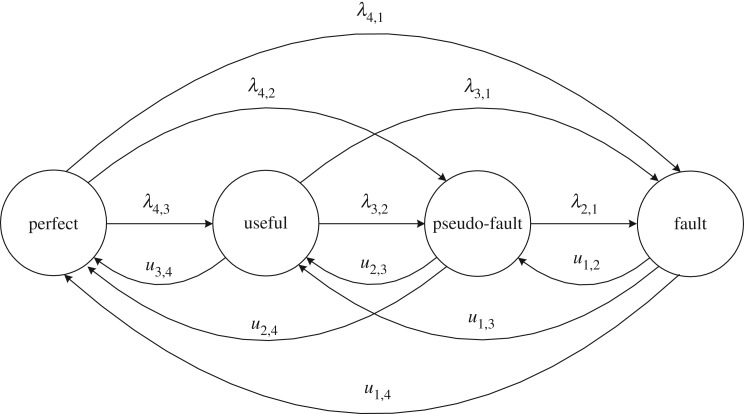
State-transition diagram for a four-state element.

Every parent node in a DBN has four states, i.e. perfect, useful, pseudo-fault and fault. The perfect state refers to perfect functioning. The state fault refers to a complete failure. In addition, the useful state and the pseudo-fault state represent the first and second degraded element states, respectively. At the beginning, each parent node in a DBN is in the perfect state. As time elapses, the DBN will either move to the useful state or the pseudo-fault state, or proceed to the fault state. For equipment that is not accessible for humans or inspection, it is only suitable to perform maintenance measures after a failure. When a non-repairable element reaches the fault state, a replacement is needed. When this happens to a repairable element, a repair is needed. The DBN can either return to the perfect state, which is viewed as a perfect repair, or it can simply return to the first or second degraded state, which is viewed as an imperfect repair. For equipment that is observable and accessible, CBM is suitable. If a state degradation occurs, the maintenance measure can be performed immediately. The element will return to the perfect state or the useful state. The state-transition diagram for an MSE is shown in figure 5. Compared with the perfect repair and imperfect repair, CBM will make the element recover from the pseudo-fault state to the perfect state or the useful state, or recover from the useful state to the perfect state. The failure rates and repair rates between the states of an element are given in a simplified mode above the state transition arcs.

Assume that at any time *t* the time interval between two consecutive time slices is Δ*t*. Then, the transition relations between the nodes in the DBN without repair, with perfect repair, with imperfect repair and under CBM can be denoted as shown in tables 2–5, respectively. The set of unacceptable states is non-negligible under CBM, which can be set as a complete failure. All state transitions from state 0 to acceptable states are not allowed. Assume that {fault} and {pseudo-fault, fault} are two absorbing sets for two different systems, and the corresponding transition relations can be denoted in tables 6 and 7, respectively.

**Table 2.** State-transition relations between states without repair.

	*t + *Δ*t*			
*t*	P	U	P-F	F
P	${\text{e}}^{-(\lambda_{4,1}+\lambda_{4,2}+\lambda_{4,3})\Delta t}$	$\frac{\lambda_{4,3}}{\lambda_{4,1}+\lambda_{4,2}+\lambda_{4,3}}$	$\frac{\lambda_{4,2}}{\lambda_{4,1}+\lambda_{4,2}+\lambda_{4,3}}$	$\frac{\lambda_{4,1}}{\lambda_{4,1}+\lambda_{4,2}+\lambda_{4,3}}$
		$\times (1-{\text{e}}^{-(\lambda_{4,1}+\lambda_{4,2}+\lambda_{4,3})\Delta t})$	$\times (1-{\text{e}}^{-(\lambda_{4,1}+\lambda_{4,2}+\lambda_{4,3})\Delta t})$	$\times (1-{\text{e}}^{-(\lambda_{4,1}+\lambda_{4,2}+\lambda_{4,3})\Delta t})$
U	0	${\text{e}}^{-(\lambda_{3,2}+\lambda_{3,1})\Delta t}$	λ3,2λ3,2+λ3,1(1−e−(λ3,2∫∫+λ3,1)Δt)	$\frac{\lambda_{3,1}}{\lambda_{3,2}+\lambda_{3,1}}(1-{\text{e}}^{-(\lambda_{3,2}+\lambda_{3,1})\Delta t})$
P-F	0	0	$e^{-\lambda_{2,1}\Delta t}$	$1-{\text{e}}^{-\lambda_{2,1}\Delta t}$
F	0	0	0	1

**Table 3.** State-transition relations between states with perfect repair.

	*t* + Δ*t*			
*t*	P	U	P-F	F
P	${\text{e}}^{-(\lambda_{4,1}+\lambda_{4,2}+\lambda_{4,3})\Delta t}$	$\frac{\lambda_{4,3}}{\lambda_{4,1}+\lambda_{4,2}+\lambda_{4,3}}$	$\frac{\lambda_{4,2}}{\lambda_{4,1}+\lambda_{4,2}+\lambda_{4,3}}$	$\frac{\lambda_{4,1}}{\lambda_{4,1}+\lambda_{4,2}+\lambda_{4,3}}$
		$\times (1-{\text{e}}^{-(\lambda_{4,1}+\lambda_{4,2}+\lambda_{4,3})\Delta t})$	$\times (1-{\text{e}}^{-(\lambda_{4,1}+\lambda_{4,2}+\lambda_{4,3})\Delta t})$	$\times (1-{\text{e}}^{-(\lambda_{4,1}+\lambda_{4,2}+\lambda_{4,3})\Delta t})$
U	0	${\text{e}}^{-(\lambda_{3,2}+\lambda_{3,1})\Delta t}$	λ3,2λ3,2+λ3,1(1−e−(λ3,2∫∫+λ3,1)Δt)	$\frac{\lambda_{3,1}}{\lambda_{3,2}+\lambda_{3,1}}(1-{\text{e}}^{-(\lambda_{3,2}+\lambda_{3,1})\Delta t})$
P-F	0	0	${\text{e}}^{-\lambda_{2,1}\Delta t}$	$1-{\text{e}}^{-\lambda_{2,1}\Delta t}$
F	$1-{\text{e}}^{-(u_{1,2}+u_{1,3}+u_{1,4})\Delta t}$	0	0	${\text{e}}^{-(u_{1,2}+u_{1,3}+u_{1,4})\Delta t}$

**Table 4.** State-transition relations between states with imperfect repair.

	*t* + Δ*t*			
*t*	P	U	P-F	F
P	${\text{e}}^{-(\lambda_{4,1}+\lambda_{4,2}+\lambda_{4,3})\Delta t}$	$\frac{\lambda_{4,3}}{\lambda_{4,1}+\lambda_{4,2}+\lambda_{4,3}}$	$\frac{\lambda_{4,2}}{\lambda_{4,1}+\lambda_{4,2}+\lambda_{4,3}}$	$\frac{\lambda_{4,1}}{\lambda_{4,1}+\lambda_{4,2}+\lambda_{4,3}}$
		$\times (1-{\text{e}}^{-(\lambda_{4,1}+\lambda_{4,2}+\lambda_{4,3})\Delta t})$	$\times (1-{\text{e}}^{-(\lambda_{4,1}+\lambda_{4,2}+\lambda_{4,3})\Delta t})$	$\times (1-{\text{e}}^{-(\lambda_{4,1}+\lambda_{4,2}+\lambda_{4,3})\Delta t})$
U	0	${\text{e}}^{-(\lambda_{3,2}+\lambda_{3,1})\Delta t}$	λ3,2λ3,2+λ3,1(1−e−(λ3,2∫∫+λ3,1)Δt)	$\frac{\lambda_{3,1}}{\lambda_{3,2}+\lambda_{3,1}}(1-{\text{e}}^{-(\lambda_{3,2}+\lambda_{3,1})\Delta t})$
P-F	0	0	${\text{e}}^{-\lambda_{2,1}\Delta t}$	$1-{\text{e}}^{-\lambda_{2,1}\Delta t}$
F	u1,4∫u1,2+u1,3+u1,4	$\frac{u_{1,3}}{u_{1,2}+u_{1,3}+u_{1,4}}$	$\frac{u_{1,2}}{u_{1,2}+u_{1,3}+u_{1,4}}$	${\text{e}}^{-(u_{1,2}+u_{1,3}+u_{1,4})\Delta t}$
	$\times (1-{\text{e}}^{-(u_{1,2}+u_{1,3}+u_{1,4})\Delta t})$	$\times (1-{\text{e}}^{-(u_{1,2}+u_{1,3}+u_{1,4})\Delta t})$	$\times (1-{\text{e}}^{-(u_{1,2}+u_{1,3}+u_{1,4})\Delta t})$

**Table 5.** State-transition relations between states under CBM.

	*t* + Δ*t*			
*t*	P	U	P-F	F
P	${\text{e}}^{-(\lambda_{4,1}+\lambda_{4,2}+\lambda_{4,3})\Delta t}$	$\frac{\lambda_{4,3}}{\lambda_{4,1}+\lambda_{4,2}+\lambda_{4,3}}$	$\frac{\lambda_{4,2}}{\lambda_{4,1}+\lambda_{4,2}+\lambda_{4,3}}$	$\frac{\lambda_{4,1}}{\lambda_{4,1}+\lambda_{4,2}+\lambda_{4,3}}$
		$\times (1-{\text{e}}^{-(\lambda_{4,1}+\lambda_{4,2}+\lambda_{4,3})\Delta t})$	$\times (1-{\text{e}}^{-(\lambda_{4,1}+\lambda_{4,2}+\lambda_{4,3})\Delta t})$	$\times (1-{\text{e}}^{-(\lambda_{4,1}+\lambda_{4,2}+\lambda_{4,3})\Delta t})$
U	$\frac{u_{3,4}}{\lambda_{3,2}+\lambda_{3,1}+u_{3,4}}$	${\text{e}}^{-(\lambda_{3,2}+\lambda_{3,1}+u_{3,4})\Delta t}$	$\frac{\lambda_{3,2}}{\lambda_{3,2}+\lambda_{3,1}+u_{3,4}}$	λ3,1∫λ3,2+λ3,1+u3,4
	$\times (1-{\text{e}}^{-(\lambda_{3,2}+\lambda_{3,1}+u_{3,4})\Delta t})$		$\times (1-{\text{e}}^{-(\lambda_{3,2}+\lambda_{3,1}+u_{3,4})\Delta t})$	$\times (1-{\text{e}}^{-(\lambda_{3,2}+\lambda_{3,1}+u_{3,4})\Delta t})$
P-F	$\frac{u_{2,4}}{\lambda_{2,1}+u_{2,3}+u_{2,4}}$	$\frac{u_{2,3}}{\lambda_{2,1}+u_{2,3}+u_{2,4}}$	${\text{e}}^{-(\lambda_{2,1}+u_{2,3}+u_{2,4})\Delta t}$	λ2,1∫λ2,1+u2,3+u2,4
	$\times (1-{\text{e}}^{-(\lambda_{2,1}+u_{2,3}+u_{2,4})\Delta t})$	$\times (1-{\text{e}}^{-(\lambda_{2,1}+u_{2,3}+u_{2,4})\Delta t})$		$\times (1-{\text{e}}^{-(\lambda_{2,1}+u_{2,3}+u_{2,4})\Delta t})$
F	u1,4∫u1,2+u1,3+u1,4	$\frac{u_{1,3}}{u_{1,2}+u_{1,3}+u_{1,4}}$	$\frac{u_{1,2}}{u_{1,2}+u_{1,3}+u_{1,4}}$	${\text{e}}^{-(u_{1,2}+u_{1,3}+u_{1,4})\Delta t}$
	$\times (1-{\text{e}}^{-(u_{1,2}+u_{1,3}+u_{1,4})\Delta t})$	$\times (1-{\text{e}}^{-(u_{1,2}+u_{1,3}+u_{1,4})\Delta t})$	$\times (1-{\text{e}}^{-(u_{1,2}+u_{1,3}+u_{1,4})\Delta t})$

**Table 6.** State-transition relations between states for absorbing set {F}.

	*t* + Δ*t*			
*T*	P	U	P-F	F
P	${\text{e}}^{-(\lambda_{4,1}+\lambda_{4,2}+\lambda_{4,3})\Delta t}$	$\frac{\lambda_{4,3}}{\lambda_{4,1}+\lambda_{4,2}+\lambda_{4,3}}$	$\frac{\lambda_{4,2}}{\lambda_{4,1}+\lambda_{4,2}+\lambda_{4,3}}$	$\frac{\lambda_{4,1}}{\lambda_{4,1}+\lambda_{4,2}+\lambda_{4,3}}$
		$\times (1-{\text{e}}^{-(\lambda_{4,1}+\lambda_{4,2}+\lambda_{4,3})\Delta t})$	$\times (1-{\text{e}}^{-(\lambda_{4,1}+\lambda_{4,2}+\lambda_{4,3})\Delta t})$	$\times (1-{\text{e}}^{-(\lambda_{4,1}+\lambda_{4,2}+\lambda_{4,3})\Delta t})$
U	$\frac{u_{3,4}}{\lambda_{3,2}+\lambda_{3,1}+u_{3,4}}$	${\text{e}}^{-(\lambda_{3,2}+\lambda_{3,1}+u_{3,4})\Delta t}$	$\frac{\lambda_{3,2}}{\lambda_{3,2}+\lambda_{3,1}+u_{3,4}}$	λ3,1∫λ3,2+λ3,1+u3,4
	$\times (1-{\text{e}}^{-(\lambda_{3,2}+\lambda_{3,1}+u_{3,4})\Delta t})$		$\times (1-{\text{e}}^{-(\lambda_{3,2}+\lambda_{3,1}+u_{3,4})\Delta t})$	$\times (1-{\text{e}}^{-(\lambda_{3,2}+\lambda_{3,1}+u_{3,4})\Delta t})$
P-F	$\frac{u_{2,4}}{\lambda_{2,1}+u_{2,3}+u_{2,4}}$	$\frac{u_{2,3}}{\lambda_{2,1}+u_{2,3}+u_{2,4}}$	${\text{e}}^{-(\lambda_{2,1}+u_{2,3}+u_{2,4})\Delta t}$	λ2,1∫λ2,1+u2,3+u2,4
	$\times (1-{\text{e}}^{-(\lambda_{2,1}+u_{2,3}+u_{2,4})\Delta t})$	$\times (1-{\text{e}}^{-(\lambda_{2,1}+u_{2,3}+u_{2,4})\Delta t})$		$\times (1-{\text{e}}^{-(\lambda_{2,1}+u_{2,3}+u_{2,4})\Delta t})$
F	0	0	0	1

**Table 7.** State-transition relations between states for absorbing set {P-F, F}.

	*t* + Δ*t*		
*t*	P	U	{P-F, F}
P	${\text{e}}^{-(\lambda_{4,1}+\lambda_{4,2}+\lambda_{4,3})\Delta t}$	$\frac{\lambda_{4,3}}{\lambda_{4,1}+\lambda_{4,2}+\lambda_{4,3}}$	$\frac{\lambda_{4,1}+\lambda_{4,2}}{\lambda_{4,1}+\lambda_{4,2}+\lambda_{4,3}}$
		$\times (1-{\text{e}}^{-(\lambda_{4,1}+\lambda_{4,2}+\lambda_{4,3})\Delta t})$	$\times (1-{\text{e}}^{-(\lambda_{4,1}+\lambda_{4,2}+\lambda_{4,3})\Delta t})$
U	u3,4∫λ3,2+λ3,1+u3,4	${\text{e}}^{-(\lambda_{3,2}+\lambda_{3,1}+u_{3,4})\Delta t}$	$\frac{\lambda_{3,2}+\lambda_{3,1}}{\lambda_{3,2}+\lambda_{3,1}+u_{3,4}}$
	$\times (1-{\text{e}}^{-(\lambda_{3,2}+\lambda_{3,1}+u_{3,4})\Delta t})$		$\times (1-{\text{e}}^{-(\lambda_{3,2}+\lambda_{3,1}+u_{3,4})\Delta t})$
{P-F, F}	0	0	1

To simplify the computation, assumptions for failure rates and repair rates of multi-state elements are made as follows:

$$\lambda_{3,1}=\lambda_{4,2},\;\lambda_{4,3}=\lambda_{3,2}=\lambda_{2,1},\;\lambda_{4,1}+\lambda_{4,2}+\lambda_{4,3}=\lambda ,\;\lambda_{4,1}:\lambda_{4,2}:\lambda_{4,3}=1:3:6$$

$$u_{1,3}=u_{2,4},u_{1,2}=u_{2,3}=u_{3,4},u_{1,2}+u_{1,3}+u_{1,4}=u,u_{1,2}:u_{1,3}:u_{1,4}=2:3:5.$$

### 3.3. Conditional probability table

If there are *n* parent nodes in a BN, and each parent node has *m* states, then *m^n^* independent parameters are needed to determine the CPTs. This is a non-deterministic polynomial (NP) problem when the number of parent nodes is large. Malcolm [32] proposed a mathematical algorithm based on the Dempster–Shafer theory and the analytic hierarchy process to determine the CPT. However, because it incorporates information from decision makers, it is computationally expensive. To solve this problem, traditional OR-gate and AND-gate constructs are introduced for the series and parallel systems. Assume that there are *n* parent nodes *X*_1_, *X*_2_, … , *X_n_* for node *Y*_,_ and the degradation probability of node *j* is *f_j_*, then the unreliability for an OR-gate can be calculated as

3.3 $$P(Y|X_{1},X_{2},\cdots ,X_{n})=1-\prod_{1\leq j\leq n}\left(1-f_{j}\right).$$

Similarly, the unreliability for an AND-gate can be expressed as

3.4 $$P(Y|X_{1},X_{2},\cdots ,X_{n})=\prod_{1\leq j\leq n}f_{j}.$$

Assume that for parent nodes A and B in figure 4, the failure rates are *λ*_A_  =  3  ×  10^−3^ and *λ*_B_  =  2  ×  10^−3^ and the repair rates are *u*_A_  =  5  ×  10^−2^ and *u*_B_  =  8  ×  10^−2^, respectively. In addition, the degradation probabilities in series and parallel systems are *P*(*C*  =  fault|A  =  useful)  =  4%, *P*(*C*  =  fault|A  = * *pseudo-fault)  =  6%, *P*(*C*  =  fault|B  =  useful)  =  2% and *P*(*C*  =  fault|B  =  pseudo-fault)  =  5%. According to the assumptions in 3.2 and in this part, the failure rates and the repair rates between the states of parent nodes A and B and the transition relations between the consecutive nodes can be obtained. When the CPTs are calculated by referring to equations (3.3) and (3.4), the reliability value of the child node C is determined, as shown in figure 4.

## 4. Case study

### 4.1. Dynamic fault tree modelling for a control unit

**Figure 6.**
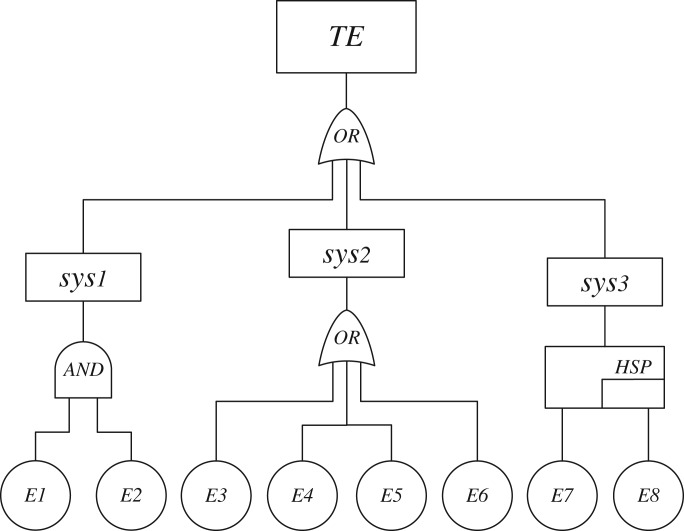
DFT model of a control unit with power in failure.

A control unit from a vibrator consisting of many electric and mechanical elements is complex and has different kinds of failure modes. In operating conditions, this control unit suffers from various environmental stresses and degrades gradually. A DFT model of the control unit is built for the case of its power in failure model, as shown in figure 6. The top event, power in failure model, is caused by three intermediate events: *sys1*, *sys2* and *sys3*. Event *sys1* contains an AND-gate with elements *E1* and *E2*. Event *sys2* contains an OR-gate with elements *E3*, *E4*, *E5* and *E6*. In addition, event *sys3* contains a hot spare gate with elements *E7* and *E8*.

### 4.2. Dynamic Bayesian network modelling for a control unit

By referring to the recorded data and consulting the domain experts, the failure rates, repair rates and degradation probabilities of the elements in the control unit are obtained and are shown in table 8.

**Table 8.** Parameters of elements in the control unit (per week).

symbol	failure rate (10^−3^)	repair rate (10^−1^)	useful (%)	pseudo_fault (%)
*E1*	4.8	1.2	4.0	6.0
*E2*	5.4	1.6	3.6	5.2
*E3*	1.0	2.6	2.0	5.6
*E4*	3.6	3.1	4.2	7.6
*E5*	3.6	3.1	4.2	7.6
*E6*	2.1	3.8	2.4	4.6
*E7*	6.0	2.6	3.8	6.4
*E8*	6.0	2.6	3.8	6.4

**Figure 7.**
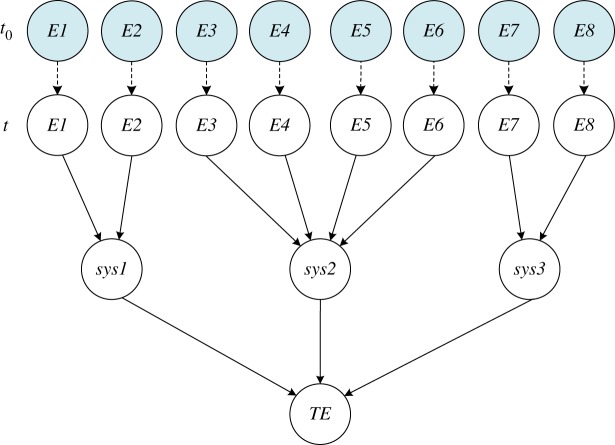
DBN model of the control unit with power in failure.

**Figure 8.**
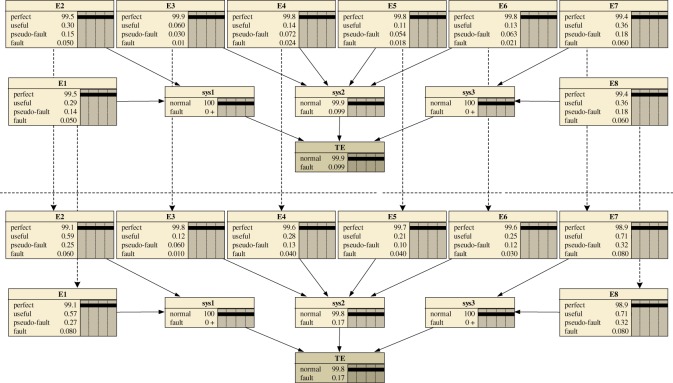
DFT model of the control unit from *t* *=* *1* to *t* *=* *2.*

Figure 7 depicts a DBN model of the control unit that was built using the algorithm to convert static and dynamic logic gates in the (dynamic) fault tree into a DBN. With the parameters provided in table 7 as inputs, a state-transition model of the control unit under CBM is established. The entire DBN model is extended from time *t*  =  1 to time *t*  =  2 as shown in figure 8. At the beginning of time, *t*  =  0, all elements are in the perfect state with a full percent. With time elapses, degradation begins.

## 5. Results and discussions

### 5.1. Model validation and reliability evaluation

**Figure 9.**
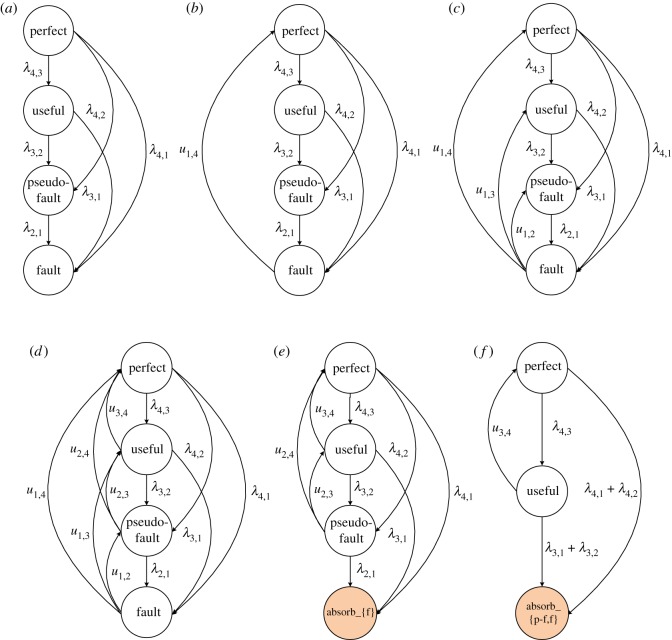
Markov processes of element *E*1 in different repair modes. (*a*) Without repair; (*b*) with perfect repair; (*c*) with imperfect repair; (*d*) under CBM; (*e*) with absorbing set {f}; (*f*) with absorbing set {p-f,f}.

**Figure 10.**
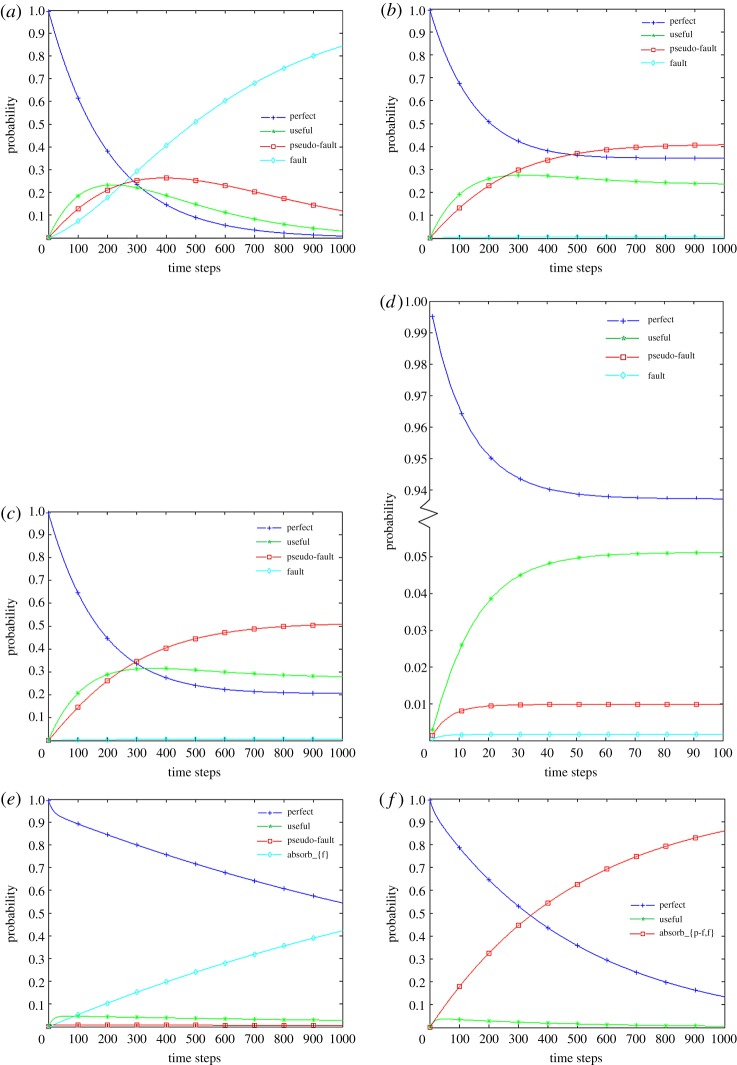
Reliability curves of element *E*1 in different repair modes. (*a*) Without repair; (*b*) with perfect repair; (*c*) with imperfect repair; (*d*) under CBM; (*e*) with absorbing set {f}; (*f*) with absorbing set {p-f,f}.

Degradation, including minor failures and major failures, can occur at any time. Let us take element *E1* in the control unit as an example. To obtain the state probabilities for the Markov process in figure 9*a*, differential equations are established in equation (5.1) according to equation (2.1). In addition, the state probability curves are drawn in figure 10*a*. It is obvious that with the increase of time steps, the probability of the perfect state drops from 1 to approximately 0 in approximately 1000 weeks. Although the probabilities of the useful state and the pseudo-fault state continue to increase for a period, the fault state captures the greatest proportion gradually.

**Figure 11.**
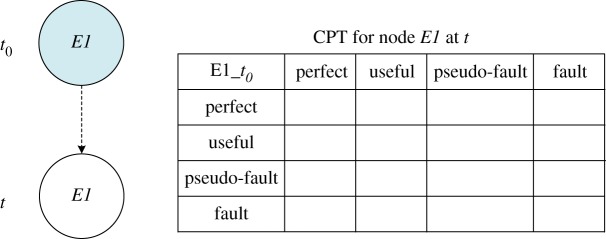
DBN model for element *E1* in different repair modes.

In figure 11, the DBN model for element *E1* at different time slices is described by a relatively simple representation, with a node at time slice *t_0_* and a node at time slice *t*. The repair mode ‘without repair’ can be denoted by using transition densities according to table 2 and figure 9*a*. An implementation of referring forward was performed with BayesiaLab software (v. 7 produced by Bayesia S.A.S. headquartered in Laval in France), and the probability curves of different states were generated that overlapped completely with the curves determined by the Markov process, which verifies the accuracy of our model.

5.1 $$\begin{array}{rl} & \frac{\text{d}p_{4}(t)}{\text{d}t}=-(\lambda_{4,3}+\lambda_{4,2}+\lambda_{4,1})p_{4}(t),\\ & \frac{\text{d}p_{3}(t)}{\text{d}t}=\lambda_{4,3}p_{4}(t)-(\lambda_{3,2}+\lambda_{3,1})p_{3}(t),\\ & \frac{\text{d}p_{2}(t)}{\text{d}t}=\lambda_{4,2}p_{4}(t)+\lambda_{3,2}p_{3}(t)-\lambda_{2,1}p_{2}(t),\\ & \frac{\text{d}p_{1}(t)}{\text{d}t}=\lambda_{4,1}p_{4}(t)+\lambda_{3,1}p_{3}(t)+\lambda_{2,1}p_{2}(t). \end{array}\}$$

The more general model is intended for repairable elements. As mentioned above, there are three kinds of repairs, namely, perfect repair, imperfect repair and CBM. In terms of perfect repair, an element can return to its perfect state from the state of failure after repair, as shown in figure 9*b*. For element *E1* in the control unit, its differential equations can be set as in equation (5.2) by referring to table 3. In terms of imperfect repair, a repair returns an element to the perfect state or one of its upper states as shown in figure 9*c*, and its differential equations can be set easily, as in equation (5.3). Under CBM, the degradation and failure can be monitored, and a repair can be performed immediately. Therefore, the system and its elements have a higher reliability and availability. By referring to equation (2.4) and table 5, the state probabilities of element *E1* can be calculated by equation (5.4) according to its Markov process in figure 9*d*. The state probability curves of element *E1* in the three kinds of repair are shown in figure 10*b–d*. Additionally, points obtained from the DBN model are drawn to verify the models.

5.2 $$\begin{array}{rl} & \frac{\text{d}p_{4}(t)}{\text{d}t}=-(\lambda_{4,3}+\lambda_{4,2}+\lambda_{4,1})p_{4}(t)+\mu_{1,4}p_{1}(t),\\ & \frac{\text{d}p_{3}(t)}{\text{d}t}=\lambda_{4,3}p_{4}(t)-(\lambda_{3,2}+\lambda_{3,1}+\mu_{3,4})p_{3}(t),\\ & \frac{\text{d}p_{2}(t)}{\text{d}t}=\lambda_{4,2}p_{4}(t)+\lambda_{3,2}p_{3}(t)-\lambda_{2,1}p_{2}(t),\\ & \frac{\text{d}p_{1}(t)}{\text{d}t}=\lambda_{4,1}p_{4}(t)+\lambda_{3,1}p_{3}(t)+\lambda_{2,1}p_{2}(t)-\mu_{1,4}p_{1}(t), \end{array}\}$$

5.3 $$\begin{array}{rl} & \frac{\text{d}p_{4}(t)}{\text{d}t}=-(\lambda_{4,3}+\lambda_{4,2}+\lambda_{4,1})p_{4}(t)+\mu_{1,4}p_{1}(t),\\ & \frac{\text{d}p_{3}(t)}{\text{d}t}=\lambda_{4,3}p_{4}(t)-(\lambda_{3,2}+\lambda_{3,1})p_{3}(t)+\mu_{1,3}p_{1}(t),\\ & \frac{\text{d}p_{2}(t)}{\text{d}t}=\lambda_{4,2}p_{4}(t)+\lambda_{3,2}p_{3}(t)-\lambda_{2,1}p_{2}(t)+\mu_{1,2}p_{1}(t),\\ & \frac{\text{d}p_{1}(t)}{\text{d}t}=\lambda_{4,1}p_{4}(t)+\lambda_{3,1}p_{3}(t)+\lambda_{2,1}p_{2}(t)-(\mu_{1,2}+\mu_{1,3}+\mu_{1,4})p_{1}(t), \end{array}\}$$

5.4 $$\begin{array}{rl} & \frac{\text{d}p_{4}(t)}{\text{d}t}=-(\lambda_{4,3}+\lambda_{4,2}+\lambda_{4,1})p_{4}(t)+\mu_{3,4}p_{3}(t)+\mu_{2,4}p_{2}(t)+\mu_{1,4}p_{1}(t),\\ & \frac{\text{d}p_{3}(t)}{\text{d}t}=\lambda_{4,3}p_{4}(t)-(\lambda_{3,2}+\lambda_{3,1}+\mu_{3,4})p_{3}(t)+\mu_{2,3}p_{2}(t)+\mu_{1,3}p_{1}(t),\\ & \frac{\text{d}p_{2}(t)}{\text{d}t}=\lambda_{4,2}p_{4}(t)+\lambda_{3,2}p_{3}(t)-(\lambda_{2,1}+\mu_{2,3}+\mu_{2,4})p_{2}(t)+\mu_{1,2}p_{1}(t),\\ & \frac{\text{d}p_{1}(t)}{\text{d}t}=\lambda_{4,1}p_{4}(t)+\lambda_{3,1}p_{3}(t)+\lambda_{2,1}p_{2}(t)-(\mu_{1,2}+\mu_{1,3}+\mu_{1,4})p_{1}(t). \end{array}\}$$

To determine the reliability of the elements, the absorbing set is introduced. There are two cases of an absorbing set, namely, the absorbing set {fault} and the absorbing set {pseudo-fault, fault}. The latter is a special case of a three-state element. The differential equations can be set according to equation (2.6) and tables 6 and 7. Considering element *E1* as an example, in the case of the absorbing set {fault}, there is no repair for the failure state shown in figure 9*e*; therefore, the state probabilities of four states can be determined by equation (5.5). In regard to the absorbing set {pseudo-fault, fault} shown in figure 9*f*, the state probabilities of the three states can be determined by equation (5.6). The state probability curves are shown in figure 9*e,f*. The state probability of the perfect state drops gradually in the absorbing set {fault}; in regard to the absorbing set {pseudo-fault, fault}, the trend becomes more obvious.

5.5 $$\begin{array}{rl} & \frac{\text{d}p_{4}(t)}{\text{d}t}=-(\lambda_{4,3}+\lambda_{4,2}+\lambda_{4,1})p_{4}(t)+\mu_{3,4}p_{3}(t)+\mu_{2,4}p_{2}(t),\\ & \frac{\text{d}p_{3}(t)}{\text{d}t}=\lambda_{4,3}p_{4}(t)-(\lambda_{3,2}+\lambda_{3,1}+\mu_{3,4})p_{3}(t)+\mu_{2,3}p_{2}(t),\\ & \frac{\text{d}p_{2}(t)}{\text{d}t}=\lambda_{4,2}p_{4}(t)+\lambda_{3,2}p_{3}(t)-(\lambda_{2,1}+\mu_{2,3}+\mu_{2,4})p_{2}(t),\\ & \frac{\text{d}p_{1}(t)}{\text{d}t}=\lambda_{4,1}p_{4}(t)+\lambda_{3,1}p_{3}(t)+\lambda_{2,1}p_{2}(t), \end{array}\}$$

5.6 $$\begin{array}{rl} & \frac{\text{d}p_{4}(t)}{\text{d}t}=-(\lambda_{4,3}+\lambda_{4,2}+\lambda_{4,1})p_{4}(t)+\mu_{3,4}p_{3}(t),\\ & \frac{\text{d}p_{3}(t)}{\text{d}t}=\lambda_{4,3}p_{4}(t)-(\lambda_{3,2}+\lambda_{3,1}+\mu_{3,4})p_{3}(t),\\ & \frac{\text{d}p_{1,2}(t)}{\text{d}t}=(\lambda_{4,1}+\lambda_{4,2})p_{4}(t)+(\lambda_{3,1}+\lambda_{3,2})p_{3}(t). \end{array}\}$$

**Figure 12.**
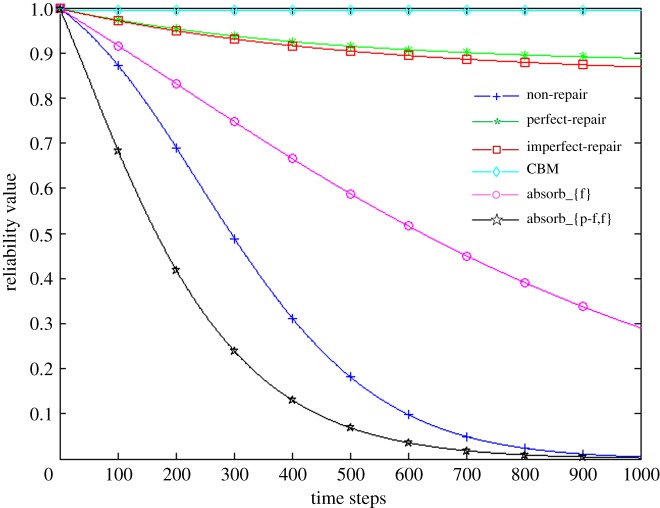
Reliability curves for the control unit.

By referring to the DBN, the reliability values of the control unit under different repair modes can be obtained, as shown in figure 12. For a control unit without the absorbing set, the CBM maintains a high reliability level of approximately 0.9947 at approximately week 1000. Compared with the imperfect repair, the perfect repair has a relatively higher reliability value. However, the imperfect repair does not affect the performance of the control unit significantly. In practice, the perfect repair will not always be attainable. For a control unit with the absorbing set, the reliability value will continue to decrease until a replacement occurs. Compared with the absorbing set {pseudo-fault, fault}, the reliability value of the control unit without repair is higher. The reliability curve of the control unit with the absorbing set {fault} more suitably reflects the degradation of elements. At approximately the 200th week, the control unit reliability remains above 0.8, and a replacement of badly degraded elements will improve its reliability significantly.

Universal generating function (UGF), another widely used reliability analysis method for an MSS, has been applied to verify the DBN model of the control unit. More details regarding UGF are available elsewhere [12,33,34]. On the basis of the Markov processes, the performance distributions of all the elements in the control unit can be determined in polynomial form. By constructing the overall model of the control unit considering its logic gates, the performance distributions of the entire MSS under the desired demand performance level are obtained through like-terms collection and a recursive procedure, which overlapped completely with the results in figure 12. Compared with that of UGF, the application of a DBN reduces a large amount of calculation and provides a more impressive result.

### 5.2. Importance analysis of the control unit

**Figure 13.**
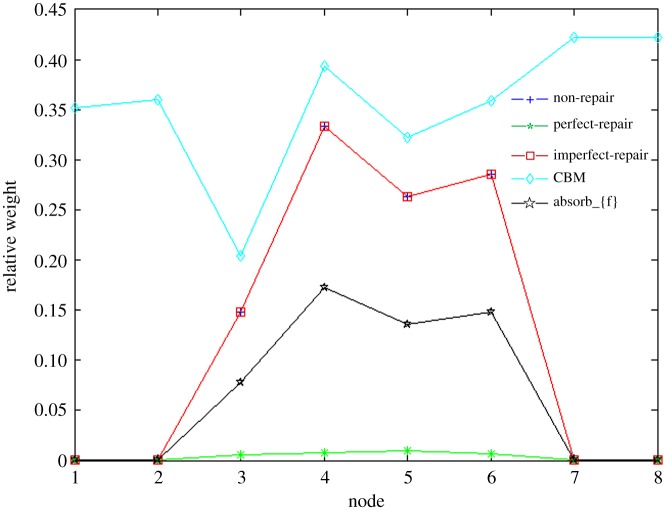
Relative weight of elements in the control unit.

The relative weights of the elements in the control unit reflect their contribution to the system performance by using mutual information, as shown in figure 13. For the control unit without repair, with imperfect repair, with perfect repair or with an absorbing set {fault}, the nodes *E3*, *E4*, *E5* and *E6*, respectively, contribute appreciably to the top event. Among them, node *E4* holds the most relative weight because it has a relatively higher failure rate. For the control unit under CBM, the repair occurs whenever a failure or degradation occurs. To maintain a stable level of high reliability, every element in the system is important. Because the failure rate of node *E3* is the lowest among the eight elements, its relative weight is lower than that of others.

## 6. Conclusion

In this paper, a method of modelling an MSS using the Markov process and a DBN is proposed, taking perfect repair, imperfect repair and CBM into account. The reliability parameter can be obtained by fusing the same parameters of elements with multi-states, and it can be predicted easily from the dynamic functions. When applying traditional methods, repetitive computation is required, which is time-intensive.

(1) Markov processes of elements without repair, with perfect repair, with imperfect repair and under CBM are established clearly, and the corresponding differential equations can be set easily.
(2) The absorbing set is non-negligible for determining the reliability of the elements and the control unit. The state probability curves of an element reflect its degradation with time, and the replacement measure can be performed at a desired level. The reliability curve of a system indicates the entire trend of its performance.
(3) Elements or the control unit under CBM can be maintained at a stable level of a higher reliability than those with perfect repair and imperfect repair.
(4) For the control unit under CBM, the reliability of all the elements should be improved. In other cases, more attention should be paid to the weak nodes, such as *E3*, *E4*, *E5* and *E6*. In reliability design and assignment, elements with a higher reliability should be taken into consideration.

## Data accessibility

Data are available from the Dryad Digital Repository: (https://doi.org/10.5061/dryad.1ch71sd) [35].
